# eIF5 and eIF5B together stimulate 48S initiation complex formation during ribosomal scanning

**DOI:** 10.1093/nar/gku877

**Published:** 2014-09-26

**Authors:** Vera P. Pisareva, Andrey V. Pisarev

**Affiliations:** Department of Cell Biology, SUNY Downstate Medical Center, 450 Clarkson Ave, Brooklyn, NY 11203, USA

## Abstract

48S initiation complex (48S IC) formation is the first stage in the eukaryotic translation process. According to the canonical mechanism, 40S ribosomal subunit binds to the 5′-end of messenger RNA (mRNA) and scans its 5′-untranslated region (5′-UTR) to the initiation codon where it forms the 48S IC. Entire process is mediated by initiation factors. Here we show that eIF5 and eIF5B together stimulate 48S IC formation influencing initiation codon selection during ribosomal scanning. Initiation on non-optimal start codons—following structured 5′-UTRs, in bad AUG context, within few nucleotides from 5′-end of mRNA and CUG start codon—is the most affected. eIF5-induced hydrolysis of eIF2-bound GTP is essential for stimulation. GTP hydrolysis increases the probability that scanning ribosomal complexes will recognize and arrest scanning at a non-optimal initiation codon. Such 48S ICs are less stable owing to dissociation of eIF2*GDP from initiator tRNA, and eIF5B is then required to stabilize the initiator tRNA in the P site of 40S subunit. Alternative model that eIF5 and eIF5B cause 43S pre-initiation complex rearrangement favoring more efficient initiation codon recognition during ribosomal scanning is equally possible. Mutational analysis of eIF1A and eIF5B revealed distinct functions of eIF5B in 48S IC formation and subunit joining.

## INTRODUCTION

Eukaryotic translation can be divided into initiation, elongation, termination and ribosomal recycling steps. Initial stage is the most regulated and, therefore, is the most complex (1,2). First, eukaryotic initiation factor (eIF) 2 binds GTP and aminoacylated initiator Met-tRNA_i_^Met^ and forms the ternary complex (TC). Next, TC associates with eIF1, eIF3 and eIF5 in the multifactor complex (MFC). MFC presence is currently confirmed in yeast, plants and mammals (3,4). Further, MFC together with eIF1A, which is the ortholog of bacterial IF1, bind 40S ribosomal subunit with the formation of 43S pre-initiation complex (43S PIC). eIF3 mediates the attachment of 43S PIC to the 5′-end of mRNA. eIF1 and eIF1A synergistically bind to the 40S subunit (5). eIF1 occupies the P site and the folded body of eIF1A binds in the A site of the 40S subunit, but its unstructured, long N- and C-terminal tails also reach into the P site (6,7). eIF1 and eIF1A together upon binding promote the rearrangement of the 40S subunit from a closed scanning-arrested to an open scanning-competent state (8). Such a conformational change underlies the mechanism by which these factors secure the fidelity of initiation codon selection. The last component of MFC, eIF5, is a GTPase-activating protein (GAP) for eIF2. eIF5 induces the hydrolysis of eIF2-bound GTP upon or after 43S PIC binding to mRNA. However, GDP and P_i_ remain associated with eIF2 until the initiation codon recognition (9).

After assembly, 43S PIC is loading onto the capped 5′-end of mRNA, employing eIFs 4A, 4B and 4F (10). eIF4F is a multi-subunit protein consisting of eIF4G (scaffold for eIF4E, eIF4A, eIF3 and some regulatory proteins), eIF4E (cap-binding protein) and eIF4A (adenosine triphosphate (ATP)-dependent RNA helicase) (11,12). 43S PIC attachment is mediated through a network of interactions involving cap, eIF4E, eIF4G, eIF3 and 40S subunit (11,13). During the attachment, eIF4A unwinds secondary structure in the 5′-untranslated region (5′-UTR) of mRNA promoting the efficient binding of the 40S subunit to the mRNA (14,15). eIF4B stimulates ATPase activity and the ATP-dependent RNA unwinding activity of eIF4A (16,17).

After mRNA attachment, 43S PIC in open state scans 5′-UTR of mRNA downstream to the initiation codon where it stops and forms 48S initiation complex (IC). eIF4A is considered to be responsible for RNA secondary structure unwinding during scanning. The majority of mammalian mRNAs contain moderately to extensively structured 5′-UTRs. 48S IC assembly on such mRNAs requires the presence of DExH box RNA helicase DHX29. This protein binds helix H16 of the 40S subunit near the mRNA entrance and promotes 48S IC formation by either direct unwinding of mRNA entering the mRNA binding channel or remodeling the ribosomal complex (18,19). The initiation codon recognition is accompanied by the establishment of mRNA codon and initiator tRNA anticodon interaction. Studies from yeast factors indicate that this interaction results in the dissociation of eIF1 and the rearrangement of 40S subunit into the closed scanning-arrested state. Notably, P_i_ release from eIF2 is the rate-limiting step in response to the initiation codon selection (9). Start codon recognition is also proposed to induce the removal of eIF1A C-terminal tail from the P-site (20). Statistical analysis in mammals revealed that the scanning complex forms 48S IC with the highest probability on AUG codons in the particular context GCC(A/G)CCAUGG, where purines at ‘−3’ and ‘+4’ positions are the most critical (21). Meanwhile, the majority of mammalian validated initiation codons deviate from the optimal context. Mainly, 43S PIC stops scanning when it encounters the first AUG codon, but, if it is in a poor context, the scanning complex may pass AUG without translation initiation (22).

Finally, 48S IC associates with the 60S ribosomal subunit with the formation of 80S IC. Subunit joining is promoted by eIF5B GTPase, an ortholog of the bacterial translation factor IF2. GTP hydrolysis by eIF5B is not required for the association activity, but is essential for its following dissociation from 80S ribosome making the latter competent for the elongation step (23). eIF5B causes the dissociation of initiation factors from the intersubunit surface of 40S subunit, which is the essential step for the ribosomal subunits association (24). eIF5B/IF2 comprises variable N-terminal and conserved central and C-terminal regions. N-terminal part is shown to be dispensable for the translation initiation (25). In contrast to eIF5B/IF2, archaeal aIF5B lacks N-terminal region and its X-ray crystal structure resembles a chalice, in which G domain (domain I), domain II and domain III form the cup, whereas domain IV founds the base. The cup and base are connected by α-helix H12 reminding the stem of the chalice. GTP binding induces conformational changes in the G domain of eIF5B which are amplified by helix H12 into a ∼5Å swing of domain IV (26). eIF1A and eIF5B compared to their bacterial orthologs have extended C-terminal ends. eIF1A possesses the long unstructured C-terminal tail (27), whereas eIF5B contains an additional α-helix H14, which is absent in IF2 (26). The interaction of eIF1A and eIF5B through their C-terminal ends is crucial for the efficient function of eIF5B in subunit joining (28). Besides the association with eIF1A, biochemical, genetic and structural analyses reveled the direct binding sites for eIF5B on the 80S ribosome. In general, eIF5B occupies the intersubunit cleft of the 80S ribosome: G domain has contacts with the 60S subunit, whereas domains II and III bind the 40S subunit (29,30). It has been proposed that eIF5B binding causes conformational rearrangements in both ribosomal subunits (30). Recent cryo-electron microscopy (cryo-EM) data shows that C-terminal domain of eIF5B upon binding to 80S ribosome changes its conformation and contacts the initiator tRNA resulting in the rearrangement and stabilization of the latter on the ribosome. Such a rearrangement also causes docking of the initiator tRNA and the initiation codon of mRNA (29). Similar stabilization of initiator tRNA by a bacterial homolog IF2 suggests the conservative mechanism of this stage during translation initiation across all kingdoms.

We investigate the mammalian translation process by reconstituting it *in vitro* from individual purified components. The position of ribosomal complexes on mRNA is analyzed by the toeprint assay. In the presence of the canonical set of initiation factors, efficient 48S ICs can be assembled only on mRNAs with single-stranded 5′-UTRs or on the β-globin mRNA comprising the short weakly structured 5′-UTR followed by the AUG start codon in the optimal nucleotide context. The 48S IC reconstitution on other cellular mRNAs is either impossible or the yield is much lower than in the rabbit reticulocyte lysate (RRL) (19; data not shown). Therefore, RRL contains activities which, probably, stabilize and improve the processivity of ribosomal complexes during the 48S IC formation. To identify the missing activities increasing the 48S IC assembly, we applied RRL fractionation with testing of intermediate fractions in the reconstituted system. Here we present that eIF5 and eIF5B together promote the 48S IC formation affecting the start codon selection during canonical ribosomal scanning. Potential mechanisms underlying such stimulation are discussed. For the first time the activity of eIF5B in the translation initiation process before and distinct from subunit joining is shown.

## MATERIALS AND METHODS

### Plasmids

See Supplementary Data.

### Purification of translation components and aminoacylation of tRNA

Native 40S subunits, DHX29, eIFs 2/3/4F and recombinant eIFs 1/1A/4A/4B/5, *Escherichia* *coli* methionyl-tRNA synthetase were purified as described (19,31). Native eIF2 βless co-purifies with eIF2 (holo form) and separates only at the last purification step on monoQ column, where it is eluted at ∼240 mM KCl. Native β-globin mRNA was purified from 10 ml RRL (Green Hectares) on poly(dT)-agarose (NEB) according to manufacturer's protocol. tRNA_i_^Met^ transcript and rabbit native total tRNAs (Promega) were aminoacylated with methionine as described (19).

### eIF5 and eIF5B purification

Native eIF5 and eIF5B were purified from RRL on the basis of activity in the stimulation of 48S IC formation *in vitro*. Purification involved preparation of ribosomal salt wash, fractionation by ammonium sulfate precipitation, chromatography on DEAE cellulose and phosphocellulose and fast protein liquid chromatography (FPLC) on MonoQ column. Recombinant eIF5B was expressed in *E. coli* and isolated by affinity chromatography on Ni-NTA agarose followed by FPLC on MonoQ column.

### Purification of eIF1A and eIF5B mutants

Recombinant eIF1A I144A, eIF5B T665A and eIF5B ΔH14 were expressed in *E. coli* and isolated by affinity chromatography on Ni-NTA agarose followed by FPLC on MonoQ column.

### Assembly of ICs

43S PIC, 48S IC and 80S IC were assembled from individual components as described (19,31). To study the GTP hydrolysis by thin-layer chromatography, 43S PIC and 48S IC were formed with [γ-32P]GTP, and 43S IC was additionally purified by sucrose density gradient (SDG) centrifugation. For methionyl-puromycin assay, we reconstituted 48S IC with 35S-labeled Met-tRNA_i_^Met^. To evaluate the effect from 4-thioU on initiation process, we compared 48S IC formation on unmodified U- and 4-thioU-containing ‘-3U’ mRNAs. In cross-linking experiments, we assembled 48S IC on co-transcriptionally 32P-labeled and 4-thioU-introduced ‘−3U’ and ‘+4U’ mRNAs. To investigate the role of eIF1A and eIF5B mutants in 80 IC formation, we employed 32P-cap-labeled β-globin mRNA.

### Toeprint assay

To evaluate the efficiency of 48S IC formation on different mRNAs and viral RNAs, we employed toeprint assay as described (19).

### GTPase assay

eIF2 TC, 43S PIC and 48S IC assembled with [γ-32P]GTP as well as [γ-32P]GTP in a free form were incubated with/without initiation factors, 40S subunits, 80S ribosomes and (CUUU)_9_ RNA (Thermo Scientific). Reaction mixtures were analyzed by chromatography on PEI cellulose as described (19).

### Purification and analysis of ribosomal complexes by SDG centrifugation

To purify assembled ribosomal complexes from unbound components, we subjected them to centrifugation through 10–30% SDG. For the composition analysis, purified complexes were resolved in sodium dodecyl sulphate-polyacrylamide gel electrophoresis (SDS-PAGE) and stained with Sypro Ruby Red (Invitrogen) or assayed by immunobloting with antibodies against eIF2α (Abcam).

### Methionyl-puromycin assay

To compare 48S ICs, assembled in the presence and in the absence of eIFs 5/5B on CAA-CUG mRNA, in the ability to form elongation-competent 80S ICs, methionyl-puromycin assay was employed. 80S ICs reconstituted with 35S-labeled Met-tRNA_i_^Met^ were treated with puromycin and extracted with ethyl acetate. 35S-labeled methionyl-puromycin formation was measured by scintillation counting of ethyl acetate extract.

### Ultraviolet (UV) cross-linking assay

To examine the role of eIF5 and eIF5B in 43S PIC rearrangement, we used UV cross-linking assay. 48S ICs assembled on 32P-labeled 4-thioU-introduced ‘−3U’ and ‘+4U’ mRNAs with/without eIFs 5/5B were irradiated at 360 nm without purification from unbound components, digested with RNAse A and assayed by SDS-PAGE and autoradiography.

### Analysis of eIF1A and eIF5B mutants activity in 80S IC formation

To test the activity of eIF1A and eIF5B mutants in subunit joining, 80S ICs were assembled on 32P-labeled β-globin mRNA with wild-type or mutant forms of eIF1A and eIF5B, and resolved by SDG centrifugation. The yield of 48S IC and 80S IC was determined by Cherenkov counting of incorporated radiolabeled mRNA in gradient fractions containing 40S subunits and 80S ribosomes, respectively.

## RESULTS

### Native eIF5 and eIF5B together are responsible for the stimulation of 48S IC formation in the reconstituted system on model mRNA with moderately structured 5′-UTR

**Figure 1.**
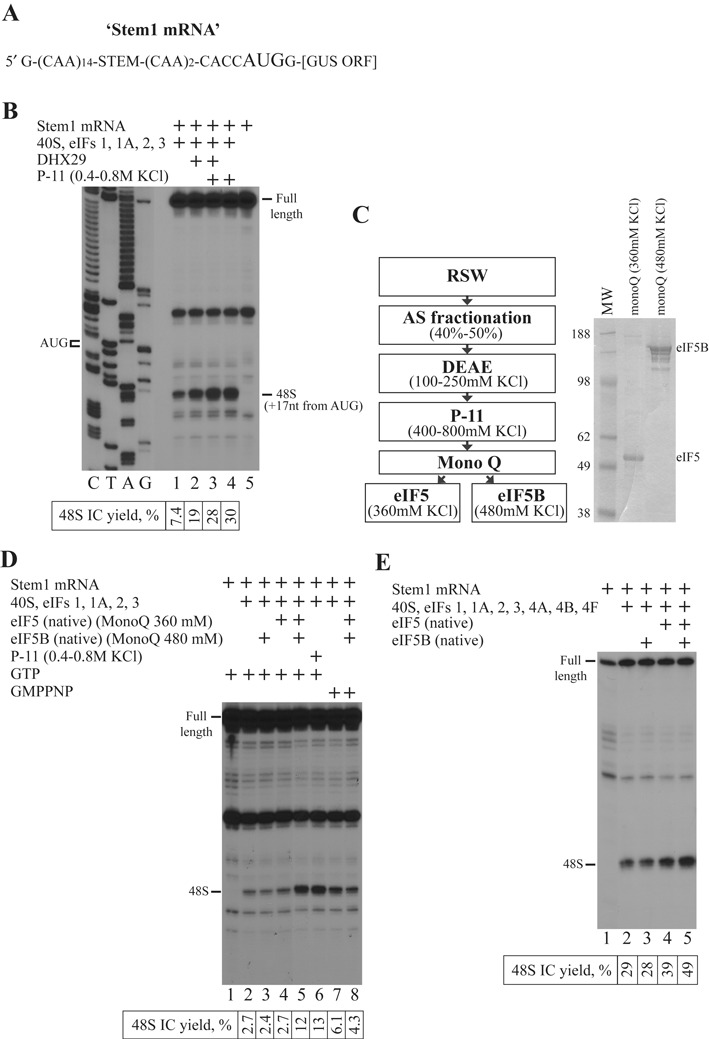
eIF5 and eIF5B are essential for stimulation of 48S IC formation. (**A**) Structure of Stem1 mRNA. (**B**, **D** and **E**) Toeprint analysis of 48S IC assembly on Stem1 mRNA in the presence of (B) RRL fraction or (D and E) different combinations of eIF5 and eIF5B. Initiation codon and position of assembled 48S IC are indicated. Lanes C/T/A/G depict corresponding DNA sequence. Toeprint assays are supplemented with quantification which shows yield of 48S IC calculated as percentage of toeprint signal to summarized signal in lane. Number of replicated experiments for quantification is at least three (*n* ≥ 3) and standard deviation is less than 14% (SD < 14%). (**C**) Left panel: purification scheme for eIF5 and eIF5B; right panel: purified eIF5 and eIF5B resolved by SDS-PAGE.

The reconstituted system comprising eIFs 1, 1A, 2, 3, 40S ribosomal subunits, Met-tRNA_i_^Met^ and DHX29 promotes a 48S IC formation on the model Stem1 mRNA consisting of single-stranded 5′-UTR with centrally introduced stem (Δ*G* = −5.5 kcal/mol) linked with β-glucuronidase open reading frame (ORF) (Figure 1A). This mRNA has been already described and imitates the one with the moderately structured 5′-UTR (19). Despite the presence of DHX29 helicase, the efficiency of 48S IC assembly in the reconstituted system on mRNAs with structured 5′-UTR is still much lower than their translation level in the RRL (19; data not shown). Therefore, to identify the activities improving the 48S yield on Stem1 mRNA, we fractionated RRL and tested fractions in the system. After several fractionation steps, we had a phosphocellulose (P-11) fraction with a dozen of proteins which stimulated 48S IC formation as revealed by a toeprint assay (Figure 1B, lanes 1–3, 5). Generally, ribosomal complexes yield toeprint signals at the position +16 to +18 nt downstream of mRNA triplet in the P site of the 40S subunits. Notably, the removal of DHX29 from the reaction did not change the efficiency of 48S IC formation (Figure 1B, lane 4). After the next fractionation step, none of fractions exhibited the activity and only the combination of two split fractions reinstated the stimulatory effect (Figure 1C, left panel). Each contributing fraction included the single protein whose fractionation scheme exactly matched the purification scheme of native eIF5 and eIF5B from RRL (Figure 1C, right panel). Native eIF5 and eIF5B synergistically but not individually promoted 48S IC formation on Stem1 mRNA with the same efficiency as the preceding phosphocellulose fraction (Figure 1D, lanes 1–6). We did not observe the effect in the presence of GMPPNP (non-hydrolysable analog of GTP) suggesting that GTP hydrolysis is essential for the stimulation (Figure 1D, lanes 7 and 8). Then, we tested the effect of native eIF5 and eIF5B in the system with the full set of initiation factors including eIFs 4A, 4B and 4F. Although the presence of eIFs 4A, 4B and 4F increased the yield of 48S IC on Stem1 mRNA (Figure 1E, lanes 1 and 2), native eIF5 and eIF5B again together but not alone improved the 48S IC formation efficiency (Figure 1E, lanes 3–5). Therefore, the mechanism of stimulation by eIF5 and eIF5B is GTP-dependent and not related to the unwinding of the 5′-UTR of mRNA.

### eIF5 and eIF5B synergistically promote 48S IC formation on model mRNAs with start codons in different nucleotide contexts

**Figure 2.**
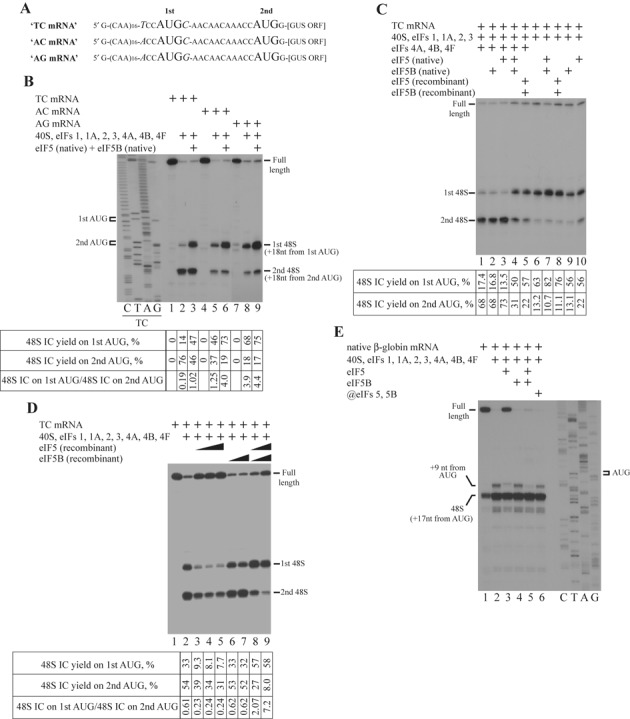
eIF5 and eIF5B stimulate 48S IC formation and support correct placement of mRNA during ribosomal scanning. (**A**) Structures of TC, AC and AG mRNAs. (**B**–**E**) Toeprint analysis of 48S IC assembly on (B) TC, AC and AG mRNAs, (C, D) TC mRNA and (E) native β-globin mRNA. Initiation codons and positions of assembled ribosomal complexes are indicated. Lanes C/T/A/G depict corresponding DNA sequences. Toeprint assays are supplemented with quantification which shows yield of 48S IC on each initiation codon calculated as percentage of toeprint signal to summarized signal in lane (*n* ≥ 3, SD < 14%).

To get insights into the mechanism of stimulation, we tested the effect of eIF5 and eIF5B in the 48S IC formation on other mRNAs whose initiation potential deviates from optimal. One subset of such mRNAs contains the initiation codon in the non-optimal initiation context. It has been shown that the scanning 43S PIC may pass the initiation codon in the non-optimal nucleotide context without the formation of the 48S IC (32). Purines in the ‘−3’ and ‘+4’ positions relative to the AUG codon are the key nucleotides in the context. We decided to test native eIF5 and eIF5B in the reconstituted system on model mRNAs comprising the single-stranded 5′-UTR followed by two initiation codons separated by the 12-nt single-stranded region (Figure 2A). The second start codon is linked with the β-glucuronidase ORF. All three mRNAs contain the second AUG codon in the optimal context, whereas the first AUG codon is in the non-optimal (TC mRNA), suboptimal (AC mRNA) or optimal (AG mRNA) context depending on the presence of two pyrimidines, one purine and one pyrimidine, or two purines in the ‘−3’ and ‘+4’ positions, respectively (32). 48S IC formed predominantly on the second AUG and weakly on the first AUG of TC mRNA (Figure 2B, lanes 1 and 2), distributed equally between both AUG codons of AC mRNA (Figure 2B, lanes 4 and 5) or assembled almost exclusively on the first AUG of AG mRNA (Figure 2B, lanes 7 and 8). Native eIF5 and eIF5B together resulted in the higher yield of 48S IC on the first codon of all three mRNAs (Figure 2B, lanes 3, 6 and 9). The highest stimulation was noted in the case of non-optimal context of the first codon presented in the TC mRNA. Therefore, we chose the TC mRNA over other mRNAs for the subsequent experiments.

To confirm the correct identification of responsible factors, we compared native eIF5 and eIF5B with their recombinant forms. We used the full-length recombinant eIF5 and the N-terminal truncated recombinant eIF5B (lacked the first 586 aa), which are both shown to be active in the subunit joining (23). We found that recombinant eIF5 and eIF5B together promote 48S IC formation as efficient as native ones (Figure 2C, lanes 1–5). Based on the reconstitution experiments on Stem1 mRNA, we found that the stimulatory effect of eIF5 and eIF5B does not depend on the presence of helicases. To confirm our finding, we assembled 48S IC in the absence of eIFs 4A, 4B and 4F on TC mRNA. It has been shown that the simultaneous omission of eIFs 4A, 4B and 4F from the reaction mixture in the reconstituted system substantially reduces the processivity of scanning ribosomes resulting in the formation of 48S IC predominantly on the first AUG codon of mRNAs with single-stranded 5′-UTRs (33). Consistently, 48S IC formed almost exclusively on the first codon of TC mRNA (Figure 2C, lane 6). Again, both native and recombinant eIF5 and eIF5B together, but not alone, promoted already efficient 48S IC formation on the first codon of TC mRNA (Figure 2C, lanes 7–10) confirming that the stimulatory effect does not depend on helicases. Interestingly, in the presence of eIF5 alone, we observed higher yield of 48S ICs on the second AUG codon (Figure 2C, lane 10), which could reflect their greater stability following eIF2*GDP release.

In the next experiment, we assayed the influence of concentration of eIF5 and eIF5B (here and thereafter we used exclusively recombinant forms of these proteins) in the stimulation. eIF5B alone at different concentrations in the presence of the whole set of canonical initiation factors did not affect the efficiency of 48S IC formation on both start codons of TC mRNA (Figure 2D, lanes 1, 2, 6 and 7). eIF5 alone in the same system reduced the yield of 48S ICs on both codons (Figure 2D, lanes 3–5). Most likely, in the absence of eIF5B, eIF5 destabilizes 48S ICs on both codons allowing GTP hydrolysis to occur followed by a eIF2*GDP release. The simultaneous increase in the concentration of both factors decreased the 48S IC assembly on the second AUG codon, but still stimulated on the first one (Figure 2D, lanes 8 and 9). Importantly, the increase of full-length signal in addition to enhancing of the 48S IC assembly on the first AUG codon of TC mRNA in the presence of the combination of eIFs 5 and 5B shown in Figure 2C and D could be explained by leaky scanning through both AUG codons. Therefore, the simultaneous rise of full-length and first AUG codon toeprint signals unambiguously indicates the reduction of 48S IC yield on the second AUG codon of TC mRNA.

### eIF5 and eIF5B eliminate the aberrant +9 nt toeprint at the stage of ribosomal scanning

48S IC assembled in the reconstituted system yields the toeprint at the position +16 to +18 downstream of mRNA triplet in the P site of the 40S subunits corresponding to the leading edge of 40S subunit on the mRNA. In addition to a major toeprint at the position +16 to +18 nt, there is also a minor one at the position +9 nt downstream of mRNA triplet in the P site. The latter is related to the 48S IC in which mRNA is not properly placed in the mRNA binding channel of 40S subunit allowing the reverse transcriptase to penetrate closer to the P site (19). Consistently, in the absence of eIF5 and eIF5B we detected +9 nt toeprint for the 48S IC assembled on the native β-globin mRNA (Figure 2E, lanes 1 and 2). eIF5B alone did not change the position and yield of this toeprint (Figure 2E, lane 4). eIF5 alone promoted a destabilization of 48S IC manifested as an increase of full-length signal intensity (Figure 2E, lane 3). The aberrant +9 nt toeprint, related to the eIF5-treated ribosomal complex, shifted 1 nt upstream of mRNA and decreased in the intensity (Figure 2E, lane 3) indicating that such an aberrant 48S IC possesses the reduced stability, so the reverse transcriptase may penetrate 1 nt close to the P site and even dissociate this complex. Notably, eIF5 and eIF5B together completely inhibited +9 nt toeprint (Figure 2E, lane 5). Importantly, in the presence of eIFs 5 and 5B, the +9 nt toeprint disappears but the full-length signal does not appear instead (Figure 2E, compare lanes 2, 3 and 5). Surprisingly, the delayed addition of eIF5 and eIF5B only slightly inhibited the +9 nt toeprint on native β-globin mRNA (Figure 2E, lane 6).

### eIF5-induced hydrolysis of eIF2-bound GTP is essential for the stimulatory activity of eIF5 and eIF5B

**Figure 3.**
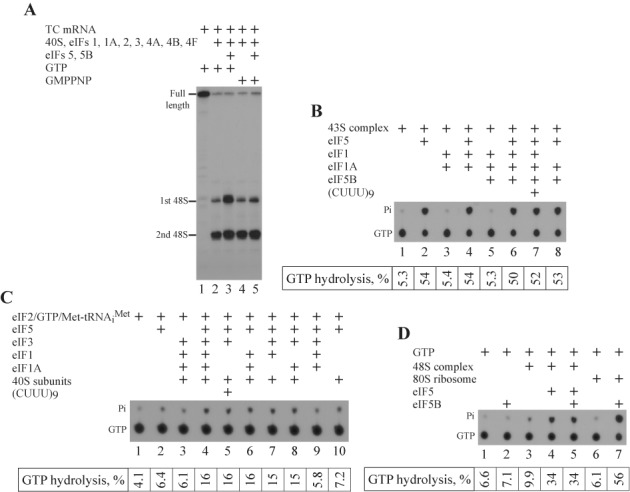
eIF5-induced hydrolysis of eIF2-bound GTP is necessary for stimulatory effect. (**A**) Toeprint analysis of 48S IC formation on TC mRNA in the presence of GTP or GMPPNP. Positions of full-length signal and assembled 48S IC are indicated. (**B**–**D**) TLC analysis of [γ^32^P]GTP hydrolysis in (B) SDG-purified 43S PIC, (C) eIF2 TC in the presence of free [γ^32^P]GTP without SDG purification and (D) 48S IC in the presence of free [γ^32^P]GTP without SDG purification by different combinations of reconstituted system components. Positions of [γ^32^P]GTP and [^32^P]Pi are indicated. TLC assays are supplemented with quantification (*n* ≥ 3, SD < 16%).

Although GTP hydrolysis is not critical for the 48S IC formation in the reconstituted system, it is absolutely necessary for the observed stimulation. The full set of initiation factors resulted in the same distribution of 48S ICs between AUG codons in the presence of GTP or GMPPNP on the TC mRNA (Figure 3A, lanes 1, 2 and 4). However, eIF5 and eIF5B revealed the stimulatory effect with GTP rather than GMPPNP (Figure 3A, lanes 3 and 5). There are two GTPases in the system—eIF2 and eIF5B. It is known that the eIF5 may induce the hydrolysis of eIF2-bound GTP before and after the 48S IC formation (9,24). eIF5B hydrolyzes GTP after subunit joining in the 80S ribosome-dependent manner. With the newly found role for eIF5B, GTP hydrolysis by this protein during the stimulatory process cannot be excluded. To determine the protein responsible for the GTP hydrolysis in the stimulatory process, we performed several GTPase assays. First, we reconstituted the 43S PIC from 40S subunits, eIFs 1, 1A, 2, 3 and [γ^32^P]-GTP, and purified it from unbound components by the centrifugation in the SDG. Then, we incubated the purified 43S PIC with different combinations of factors and examined the GTP hydrolysis by the thin layer chromatography (TLC). 43S PIC alone or supplemented with eIFs 1 and 1A together (since eIF1A dissociates from 40S subunit during SDG centrifugation (30) and, probably, affects eIF1 association with 43S PIC due to cooperative binding) did not cause the GTP hydrolysis (Figure 3B, lanes 1 and 3). The addition of eIF5B to the 43S PIC supplemented with eIFs 1 and 1A did not change the result (Figure 3B, lane 5). As expected, the incubation of 43S PIC with eIF5 yielded the efficient GTP hydrolysis (Figure 3B, lane 2). The presence of additional eIFs 1 and 1A together (Figure 3B, lane 4) or in the combination with eIF5B (Figure 3B, lane 6) did not influence the efficiency of eIF5-induced hydrolysis after 15 min of incubation. The addition of eIF1A/eIF5B complex to the 43S PIC with eIF5 also did not influence the efficient GTP hydrolysis (Figure 3B, lane 8). 43S PIC supplemented with the single-stranded RNA, which occupies the mRNA binding channel of the 40S subunit, imitates the scanning ribosomal complexes. To compare the GTP hydrolysis in the 43S PIC and the scanning ribosomal complex, we incubated 43S PIC supplemented with eIFs 1, 1A, 5, 5B in the absence or presence of single-stranded (CUUU)_9_ RNA and found that GTP hydrolysis was similar in both cases (Figure 3B, lanes 7 and 8). We would like to point out that we measured the thermodynamics of GTP hydrolysis after 15 min of incubation of reactions rather than the kinetics as described in the previous report on the mammalian reconstituted system by Pestova *et al.* (24). In that report eIF1 reduces the hydrolysis rate, but the yield of GTP hydrolysis induced by eIF5 in the presence and in the absence of eIF1 after 15 min of 43S PIC incubation is very similar. Therefore, our results do not contradict reported data. In conclusion, the yield of GTP hydrolysis by eIF2 is equally efficient in the 43S PIC and in the scanning ribosomal complex, and not affected by eIFs 1, 1A and 5B.

To obtain details of GTP hydrolysis in the 43S PIC, we assayed the role of individual components in the process. Importantly, in contrast to the experiment shown in Figure 3B, this and next experiments on GTP hydrolysis were conducted in the presence of free [γ^32^P]-GTP without the SDG purification step. As a result, the yield of GTP hydrolysis was lower. We found that eIF5 and 40S subunit were essential for the hydrolysis (Figure 3C, lanes 1–3 and 9). On the other hand, eIF2 TC in the presence of eIF5 and 40S, but in the absence of eIFs 1, 1A and 3, could not attach the 40S subunit and hydrolyze GTP (Figure 3C, lane 10). Different combinations of eIFs 1, 1A and 3 promoting the association of eIF2 TC with the 40S subunit yielded the GTP hydrolysis in the presence of eIF5 (Figure 3C, lanes 4 and 6–8). It has been shown that the combination of eIF3 and single-stranded RNA stimulates binding of eIF2 TC to the 40S subunit (34). Consistently, the employment of eIF3 and single-stranded (CUUU)_9_ RNA for the attachment of eIF2 TC to the 40S subunit also caused GTP hydrolysis in the presence of eIF5 (Figure 3C, lane 5). Therefore, the attachment of eIF2 TC to the 40S subunit is critical for the eIF5-induced hydrolysis of GTP.

We found that eIF5B does not hydrolyze GTP during the 43S PIC formation and the following ribosomal scanning processes. Next, we assayed the GTPase activity of eIF5B in the entire process when eIF5 and eIF5B synergistically stimulate the 48S IC formation. Despite eIF5B efficiently hydrolyzed GTP in the presence of 80S ribosomes in the control reaction (Figure 3D, lanes 1, 2, 6 and 7), it did not reveal the GTPase activity during the stimulation of the 48S IC formation and all hydrolyzed GTP was related to eIF2 (Figure 3D, lanes 3–5). Since the presence of eIF5B does not change the yield of GTP hydrolysis after 43S PIC formation (Figure 3B) and during 48S IC assembly (Figure 3D), we conclude that GTP hydrolysis by eIF5B is not essential for the stimulatory effect.

### α and β subunits of eIF2 as well as nucleotide modifications of initiator tRNA are not essential for the stimulatory effect

**Figure 4.**
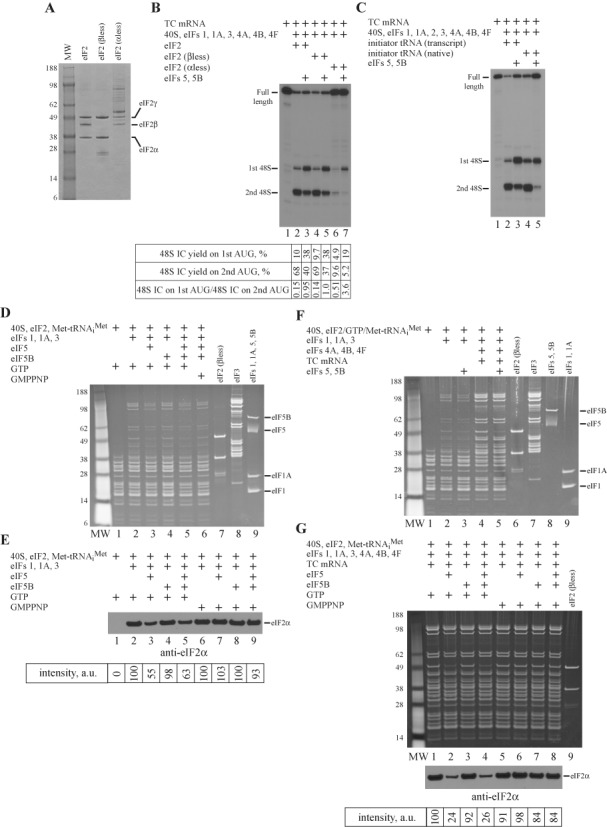
Role of eIF2 and initiator tRNA in stimulation; effect of eIF5 and eIF5B on 43S PIC versus 48S IC composition. (**A**) Different forms of eIF2 assayed by SDS-PAGE. (**B** and **C**) Toeprint analysis of 48S IC formation on TC mRNA in the presence of different forms of (B) eIF2 (supplemented with quantification; *n* = 3, SD < 14%) and (C) initiator tRNA. Positions of full-length signal and assembled 48S IC are indicated. (**D–G**) Influence of eIF5 and eIF5B on the composition of (D and E) 43S PIC, (F) 43S PIC versus 48S IC and (G) 48S IC assayed by SDG centrifugation and SDS-PAGE (D, F and G, top panel) or immunoblotting with antibodies against eIF2α (E and G, bottom panel). Immunoblotting assays are supplemented with quantification (*n* ≥ 3, SD < 15%). Intensity of eIF2α signal is shown in arbitrary units (a.u.).

Stimulation of 48S IC formation strictly depends on the GTP hydrolysis by eIF2, which consists of α, β and γ subunits. eIF2γ is involved in the initiator tRNA recruitment to the 40S ribosomal subunit and GTP hydrolysis, eIF2β interacts with other initiation factors eIF5 and eIF2B, whereas eIF2α plays the regulatory role in the activity of eIF2 (2). αless and βless forms of eIF2 could be purified from RRL along with the holo form of protein (Figure 4A). To test the role of individual subunits in the process, we replaced the holo form of eIF2 in the system with its αless or βless forms. We found that the holo and βless forms of eIF2 contribute equally to the yield of 48S IC, which is consistent with the reported data in mammalian reconstituted system (32), and to the stimulatory effect (Figure 4B, lanes 1–5). It is shown that α-subunit of eIF2 is responsible for the recognition of ‘−3’ context position by scanning ribosomal complexes (32). Consistently, the substitution eIF2 holo form for αless form reduced the yield of 48S IC formation on both initiation codons, but did not change the stimulatory effect (Figure 4B, lanes 6 and 7). Obtained data suggest that α and β subunits of eIF2 are not necessary for the stimulatory process.

Initiator Met-tRNA_i_^Met^ along with eIF2 and GTP are the components of eIF2 TC. Nucleotide modifications of tRNA result in the fine tuning of molecular structure for the optimal functional performance and, therefore, play an important role in the translation (35). To examine the role of nucleotide modifications in the stimulation, we compared native and transcript forms of initiator tRNA in the system. eIF5 and eIF5B stimulated the 48S IC formation on the first AUG codon of TC mRNA in the presence of either form suggesting that nucleotide modifications of initiator tRNA are not essential for the effect (Figure 4C).

### Influence of eIF5 and eIF5B on the composition of 43S PIC

Since the stimulation takes place during ribosomal scanning, to get insights into the mechanism of the stimulatory effect, we examined the potential changes in the composition of 43S PIC in the presence of eIF5 and eIF5B. Although the roles of eIFs 5 and 5B in dissociating eIF2*GDP following AUG recognition have been published (24), the effect of these proteins on the integrity of 43S PIC has not been reported. For the analysis, we reconstituted the 43S PIC with different combinations of eIF5 and eIF5B, and purified it by the SDG centrifugation. Due to the high adsorption of proteins to the gradient tube, the experiment required the large amounts of initiation factors. Therefore, we replaced the eIF2 holo form in the reaction with the βless form, since they are both active in the stimulation. We found that in the presence of GTP, eIF2 and eIF3 associate with the 40S subunit in the purified 43S PIC (Figure 4D, lane 2). eIF2 TC alone does not interact with the 40S subunit (Figure 4D, lane 1). eIF5-induced hydrolysis of eIF2-bound GTP reduces the affinity of eIF2 and eIF3, which associate cooperatively, to the 43S PIC (Figure 4D, lane 3). Interestingly, eIF5B alone or in the combination with eIF5 does not influence the integrity of the 43S PIC (Figure 4D, lanes 4 and 5). Consistently, eIF5 and eIF5B together in the presence of non-hydrolysable GMPPNP has no effect on the 43S PIC (Figure 4D, lane 6). To support our findings, we also calculated the affinity of eIF2 in different 43S PICs. Due to the low signal from eIF2 in the 43S PIC, the quantification was based on the immunoblotting assay (Figure 4E).

Compare to 43S PIC, eIF2 and eIF3 bind with the higher affinity to the 48S IC (Figure 4F, lanes 1, 2 and 4). It is well known that mRNA in the mRNA binding channel of the 40S subunit contributes to the cooperative association of factors (34). In contrast to the 43S PIC (Figure 4F, lanes 2 and 3), eIF5 and eIF5B together in the presence of GTP substantially reduce the affinity of eIF2 but does not change the affinity of eIF3 to the 48S IC (Figure 4F, lanes 4 and 5). To match data on 43S PIC and 48S IC, we compared the effects of eIF5 alone and in combination with eIF5B in the presence of GTP versus GMPPNP on the integrity of 48S IC (Figure 4G, top panel), and calculated the affinity of eIF2 in different 48S ICs employing the immunoblotting assay (Figure 4G, bottom panel). The obtained data suggest that eIF5 alone causes the dissociation of eIF2 from both 43S PIC and 48S IC in the presence of GTP rather than GMPPNP, whereas eIF5B has no effect on the integrity of complexes (Figure 4D–G). The stronger effect of eIF5 on the affinity of eIF2 to 48S IC rather than 43S PIC is unambiguously explained by the existence of base pairing of Met-tRNA_i_^Met^ with the AUG codon of the TC mRNA stabilizing eIF2 association with the 48S IC compared to the 43S PIC (Figure 4F, compare lanes 2 and 4). Consistently, GTP hydrolysis leads to the disruption of the contacts between eIF2 and base-paired Met-tRNA_i_^Met^ resulting in the greater dissociation of eIF2 from 48S IC than from 43S PIC. We would like to stress again that we measured thermodynamic rather than kinetic effects. In agreement with the data reported by Pestova *et al.* on mammalian reconstituted system (24), the yield of eIF5-induced hydrolysis of eIF2-bound GTP in the presence and in the absence of AUG codon after 15 min of incubation of 43S PIC and 48S IC, respectively, is very similar. Therefore, the difference in the association of eIF2 with 43S PIC and 48S IC after the eIF5-induced hydrolysis of eIF2-bound GTP cannot be explained by the change in the GTP hydrolysis rate. It has been shown that eIF5 and eIF5B together after the establishment of codon–anticodon interaction almost completely dissociate eIF2 from the 40S subunit (32), whereas eIF3, due to additional contacts with mRNA, dissociates only after 80S IC formation (24). Therefore, our data are in a good agreement with the published results.

Although eIF5 and eIF5B play the important role in the stimulation process, we did not find their association with the 43S PIC and the 48S IC (Figure 4D and F). So, we conclude that they have a low affinity to the 43S PIC. Since eIF1A and, probably, eIF1 dissociate from 43S PIC during centrifugation, we examined the interplay between eIFs 1, 1A and eIFs 5, 5B in the different assay.

### Interplay between eIFs 5, 5B and eIFs 1, 1A in the stimulatory process

**Figure 5.**
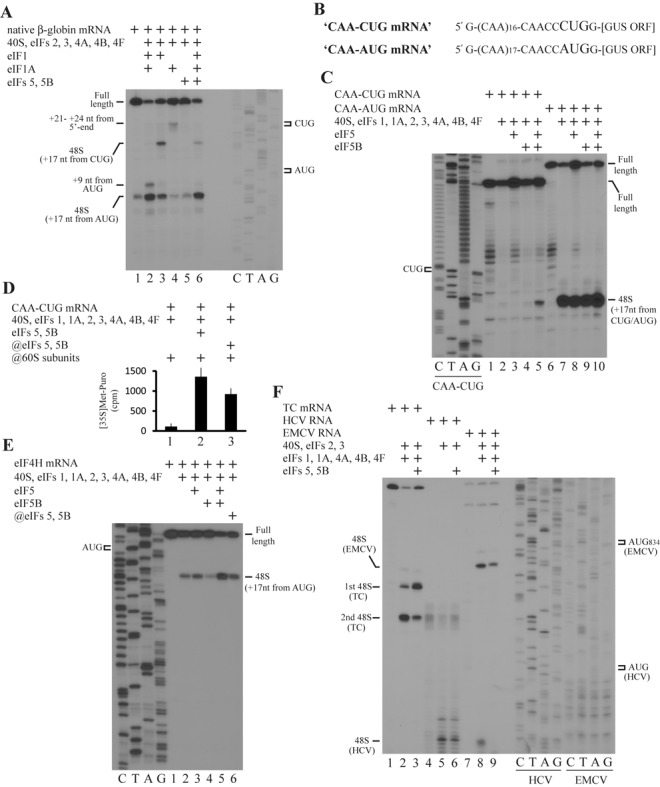
Role of eIF5 and eIF5B in 48S IC formation on different RNAs. (**A**, **C**, **E**, **F**) Toeprint analysis of 48S IC assembly on (A) native β-globin mRNA, (C) CAA-CUG and CAA-AUG mRNAs, (E) eIF4H mRNA and (F) TC mRNA, HCV and EMCV RNAs in the presence of different combinations of eIF5 and eIF5B. Initiation codons and positions of assembled 48S IC are indicated. Lanes C/T/A/G depict corresponding DNA sequences. (**B**) Structures of CAA-CUG and CAA-AUG mRNAs. (**D**) Methionyl-puromycin assay of 80S IC formation on CAA-CUG mRNA.

eIF1 and eIF1A are not necessary for 48S IC formation on mRNAs with unstructured 5′-UTRs (19,33). In contrast, 48S IC assembly on mRNAs with even weakly structured 5′-UTRs strictly requires the simultaneous presence of both factors (33,36). So far, all reconstitution experiments were mostly conducted on model mRNAs with unstructured 5′-UTRs. Therefore, we could not evaluate the role of eIF1 and eIF1A in the stimulatory effect. To examine the potential interplay between eIFs 5, 5B and eIFs 1, 1A in the process, we performed the reconstitution on a native β-globin mRNA, which represents the mRNA with the low structured 5′-UTR. It has been shown that 43S PIC is arrested at different characteristic positions along the β-globin mRNA during ribosomal scanning in the absence of eIF1 or eIF1A (33,36). Since the reconstitution yield is very high on β-globin mRNA, for better discrimination of potential effects we decided to reduce 2-fold the concentration of initiation factors in the system. In our experiments the presence of the full set of canonical initiation factors results in the efficient 48S IC formation on the AUG start codon of the β-globin mRNA (Figure 5A, lane 2). Note the background signal at the same position on the mRNA in the absence of factors (Figure 5A, lane 1). The omission of eIF1A causes the 48S IC assembly on both the AUG start codon and the preceding near-cognate CUG codon (Figure 5A, lane 3). The removal of eIF1 has even more dramatic effect. Consistently with described results (33,36), 43S PIC in the absence of eIF1 can attach to the mRNA but cannot scan downstream yielding the toeprint at the position +21 to +24 nt from the 5′-end of mRNA (Figure 5A, lane 4), whereas the simultaneous omission of both factors leads to no toeprints (Figure 5A, lane 5). The presence of both eIF5 and eIF5B with the full set of canonical initiation factors does not change the efficiency of 48S IC formation on the AUG start codon and promotes very inefficient 48S IC assembly on the preceding non-cognate CUG codon suggesting that eIF5 and eIF5B do not influence the affinity of eIF1 and eIF1A to the 43S PIC (Figure 5A, lane 6).

### eIF5 and eIF5B stimulate 48S IC formation on mRNAs with CUG and 5′-end located AUG start codons

In eukaryotes, AUG is not the only start codon and translation initiation may take place on different codons. In mammals CUG appears to be the most efficient non-AUG initiation codon. In general, non-AUG initiation results in a longer protein isoform in addition to an isoform obtained from the initiation on the standard downstream located AUG start codon. It has been suggested that non-AUG initiation plays the regulatory role in the eukaryotic cells (37). Since the presence of optimal nucleotide context is critical for the initiation on non-AUG codons (38), we employed CAA-CUG and CAA-AUG model mRNAs comprising single-stranded 5′-UTR followed by CUG or AUG initiation codon in a good nucleotide context, respectively, linked with β-glucuronidase ORF (Figure 5B). The incubation of the CAA-CUG mRNA with the canonical set of initiation factors results in the inefficient 48S IC formation on the CUG start codon (Figure 5C, lanes 1 and 2). The presence of eIF5 inhibits the 48S IC yield due to a 48S IC destabilization, whereas eIF5B has no effect (Figure 5C, lanes 3 and 4). Importantly, the simultaneous addition of eIF5 and eIF5B substantially enhances the 48S IC reconstitution (Figure 5C, lane 5). As expected, the canonical set of initiation factors gives almost an absolute 48S IC formation on the AUG start codon of CAA-AUG mRNA (Figure 5C, lanes 6 and 7). eIF5 and eIF5B individually or together do not essentially change the high yield of 48S IC (Figure 5C, lanes 8–10). To confirm that the 48S IC assembled on the CUG start codon in the presence of eIF5 and eIF5B is competent for the subsequent translation steps, we employed the methionyl-puromycin assay. The higher yield of methionyl-puromycin for the 48S IC reconstituted in the presence rather than in the absence of eIF5 and eIF5B indicates its translation competence (Figure 5D).

Infrequently, AUG start codon is located at the very 5′-end of mRNA. We analyzed mRNAs encoding translation initiation factors and found that the initiation codon in the eIF4H mRNA is situated just 9 nt downstream from the 5′-end. Interestingly, mRNA in such a 48S IC does not entirely occupy the mRNA binding channel extending ∼15 nt downstream and upstream from the codon in the P site of the 40S subunit (39). Moreover, there are no contacts between mRNA and eIF3 in this 48S IC, since eIF3 interacts with positions −8 to −17 (relative to the +1A of the AUG initiation codon) (39). It is well known that 40S-mRNA and eIF3-mRNA contacts substantially contribute to the integrity and stability of 48S IC (24). Therefore, 48S IC assembly on such AUG codons may require the additional stabilization of components in the 43S PIC during start codon recognition. Indeed, we did not observe the efficient 48S IC formation on eIF4H mRNA in the presence of the full set of canonical initiation factors in the system (Figure 5E, lanes 1 and 2). The addition of eIF5 does not have any effect (Figure 5E, lane 3), whereas the presence of eIF5B even slightly inhibits the 48S IC assembly (Figure 5E, lane 4). Nevertheless, eIF5 and eIF5B together appreciably stimulate the 48S IC formation (Figure 5E, lane 5). Notably, a delayed addition of these factors increases a little the 48S IC yield as a result of additional *de novo* reconstitution (Figure 5E, lane 6).

### eIF5 and eIF5B do not show the stimulatory effect in translation initiation mediated by internal ribosome entry site (IRES) mechanism

Although the 48S IC formation on cellular mRNAs occurs by ribosomal scanning, the initiation on some viral RNAs relies on the IRES-dependent mechanism, when 40S subunit binds to structural elements within mRNA in such a way that AUG start codon directly enters the P site or is located in its close proximity. Internal initiation often requires the limited set of factors. For instance, hepatitis C virus (HCV) RNA depends only on 40S subunit, eIF2 TC and eIF3, whereas eIF1 and eIF1A are dispensable for the initiation on EMCV RNA (40,41). To evaluate the effect of eIF5 and eIF5B in the IRES-mediated process, we performed the reconstitution on these viral RNAs. The limited set and the full set of initiation factors result in the efficient 48S IC formation on HCV and EMCV RNAs, respectively (Figure 5F, lanes 4, 5, 7 and 8). Despite eIF5 and eIF5B together stimulate 48S IC assembly on the first start codon of control TC mRNA (Figure 5F, lanes 1–3), these proteins even slightly inhibited the 48S IC yield on both viral RNAs (Figure 5F, lanes 6 and 9), suggesting that the stimulatory effect is limited by the canonical ribosomal scanning mechanism of initiation.

### eIF5 and eIF5B cause the 43S PIC rearrangement during ribosomal scanning as revealed by UV cross-linking experiments

**Figure 6.**
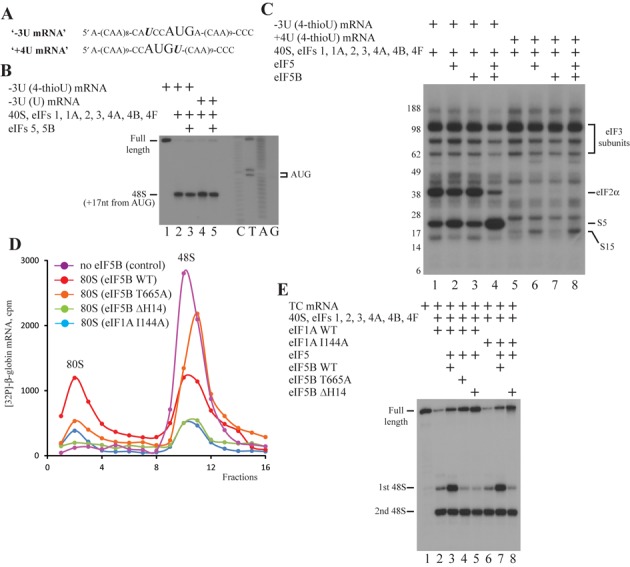
eIF5 and eIF5B cause 43S PIC rearrangement; mutational analysis of eIF1A and eIF5B. (**A**) Structures of ‘−3U’ and ‘+4U’ mRNAs. (**B**) Toeprint analysis of 48S IC formation on ‘−3U’ mRNAs containing 4-thioU and unmodified U. Initiation codon and position of assembled 48S IC are indicated. Lanes C/T/A/G depict corresponding DNA sequence. (**C**) UV cross-linking of 32P-labeled ‘−3U’ and ‘+4U’ mRNAs containing 4-thioU with components of 48S IC before and after incubation with eIF5 and eIF5B, assayed by SDS-PAGE. Positions of molecular weight markers are shown on the left. (**D**) 80S IC formation on 32P-labeled β-globin mRNA with eIF5B WT (red), eIF5B T665A (orange), eIF5B ΔH14 (green), eIF1A I144A (blue) or without eIF5B WT (magenta) assayed by SDG centrifugation. Upper fractions are omitted for clarity. (**E**) Toeprint analysis of 48S IC formation on TC mRNA in the presence of different eIF1A and eIF5B mutants. Positions of full-length signal and assembled 48S IC are indicated.

To get more insights in the stimulatory mechanism, we examined potential rearrangements within 43S PIC in the presence of eIF5 and eIF5B during ribosomal scanning employing the directed UV cross-linking assay. The technique is based on the low-energy UV irradiation of RNA with a co-transcriptionally introduced 4-thiouridine (4-thioU) resulting in the specific activation of 4-thioU for cross-linking. Since the strength of nucleotide context of start codon influences the stimulatory efficiency of eIF5 and eIF5B, we decided to use two model ‘−3U’ and ‘+4U’ mRNAs comprising (CAA) repeat-based 5′-UTR and ORF, and the locally introduced single uridine in either ‘−3’ or ‘+4’ key context position, respectively (Figure 6A). To rule out the influence of 4-thioU on the reconstitution, we employed toeprint assay. The full set of initiation factors results in the highly efficient 48S IC formation on both unmodified U- and 4-thioU-containing ‘−3U’ mRNAs (Figure 6B, lanes 1, 2 and 4) and the simultaneous presence of eIF5 and eIF5B does not change the already optimal assembly on both mRNAs (Figure 6B, lanes 3 and 5) suggesting that the 4-thioU-containing mRNA has no impact on the process. Since eIF5 and eIF5B dissociate from ribosomal complexes during SDG centrifugation, to preserve the integrity of the process we irradiated the 48S IC immediately after the assembly without the purification by SDG centrifugation. Although this approach gives the extensive background cross-links, it correctly reflects the reconstitution process. It has been demonstrated that 4-thioU at the −3 position of mRNA in the SDG-purified 48S IC cross-links with eIF2α and ribosomal protein rpS5, whereas 4-thioU at the +4 position with ribosomal protein rpS15 (32). The reconstitution assay revealed that eIF2α is directly involved in the context recognition (32). rpS15 is a part of tRNA binding pocket of P site and, therefore, may contribute to the association of initiator tRNA with the 40S subunit. Probably, it also promotes the establishment of codon–anticodon interactions by stabilizing the base-pared conformation of initiator tRNA during scanning (42). In turn, rpS5 may stimulate the 48S IC formation by the interaction with the nucleotide at the −3 position (32).

Consistently, in our experiment 4-thioU at the −3 position of co-transcriptionally radiolabeled ‘−3U’ mRNA efficiently cross-links with eIF2α and rpS5 (Figure 6C, lane 1). The addition of eIF5 alone slightly decreases cross-linking to eIF2α and increases to rpS5 (Figure 6C, lane 2). As expected, eIF5B alone has no impact (Figure 6C, lane 3). Importantly, eIF5 and eIF5B together promote almost complete dissociation of eIF2 from the 48S IC and, therefore, substantially reduce the cross-link with eIF2α, but also strongly stimulate the cross-link with rpS5 (Figure 6C, lane 4). It has been reported that eIFs 5 and 5B together promote almost complete dissociation of eIF2 from reconstituted mammalian 48S IC which is not purified through SDG centrifugation (32). Therefore, our results are in a good agreement with the reported data. In turn, 4-thioU at the +4 position of ‘+4U’ mRNA in the 48S IC weakly cross-links with rpS15 (Figure 6C, lane 5). The supplementation of reaction with eIF5 alone enhances cross-linking with rpS15 (Figure 6C, lane 6). Although eIF5B alone has no effect (Figure 6C, lane 7), eIF5 and eIF5B together increase cross-linking with rpS15 even more than eIF5 alone does (Figure 6C, lane 8). Since we followed the cross-linking protocol essentially as described (32) and omitted only the step of ribosomal complex purification through the SDG centrifugation, the assignment of rpS5, rpS25 and eIF2α was made by matching our results and reported gel data. The equal intensity of cross-links to eIF3 subunits indicate that the same amount of 48S IC is loaded in the each lane (Figure 6C, lanes 1–8). In conclusion, the increase of cross-links to rpS5 and rpS15 in −3 and +4 positions indicates the 40S/mRNA rearrangement within the decoding center of small ribosomal subunit, which should contribute to the stimulatory effect.

### Mutational analysis of eIF1A and eIF5B split functions of eIF5B in the 48S IC formation and subunit joining

During subunit joining, the main function of eIF5B is the clearance of the intersubunit area of the 40S subunit from the initiation factors for the following association of small and large ribosomal subunits. However, this role of eIF5B is destructive for the 43S PIC and may not take place during ribosomal scanning. On the other hand, some eIF5B activities, like the recently found stabilization of Met-tRNA_i_^Met^ in the P site (29), may be attributed to both steps. So, there should be a switch between eIF5B functions.

To test our suggestion, we constructed one mutant of eIF1A (eIF1A I144A) and two mutants of eIF5B (eIF5B ΔH14 and eIF5B T665A). It has been shown that eIF1A and eIF5B interact through C-terminal tails and this contact is essential for the subunit joining. Particularly, yeast eIF1A I153A or eIF5B ΔH14 mutants containing a substitution and a deletion at the very end of C-terminal tails, respectively, independently reduce both GTP hydrolysis and subunit joining activities of eIF5B *in vitro* (28). At the same time, yeast eIF1A I153A is as active in the preceding steps of translation initiation as the wild-type form (28). Therefore, eIF1A I144A and eIF5B ΔH14 are human analogs of these yeast mutants. It has been also described that yeast eIF5B T439A mutant with the substitution in the GTP-binding center is unable to coordinate Mg^2+^-ion in the active center and hydrolyze GTP. As a result, this mutant still promotes subunit joining but cannot dissociate from the 80S IC (43). So, human eIF5B T665A is the analog of yeast eIF5B T439A.

We tested the functional activity of mutants in subunit joining. For that, we reconstituted 48S IC on radiolabeled β-globin mRNA transcript from the full set of initiation factors, supplemented the reaction with eIF5, eIF5B and 60S subunits, and separated the ribosomal complexes by a SDG centrifugation. Importantly, we reduced 2-fold the amount of eIF5 end eIF5B forms for better discrimination of effects. Thus, in the presence of eIF5 and wild-type eIF5B, 48S IC forms 80S IC with the efficiency close to 50% (Figure 6D, red line). The omission of eIF5B completely inhibits the 80S IC formation (Figure 6D, magenta line). As expected, eIF5 and eIF5B T665A also promote the 80S IC assembly, but with the lower efficiency than wild-type proteins (Figure 6D, orange line). When we replaced wild-type eIF5B with eIF5B ΔH14 or wild-type eIF1A with eIF1A I144A, we observed severe impairment of 80S IC formation and, unexpectedly, strong dissociation of 48S IC (Figure 6D, green and blue lines). This finding implies that eIF5B initiates subunit joining by preparing a 48S IC, but cannot complete it in the absence of a checkpoint contact with eIF1A resulting in a 48S IC disassembly. Therefore, all tested protein mutants are functional.

We examined our mutants in the ability to stimulate 48S IC formation on TC mRNA. eIF5 in the presence of eIF5B ΔH14 (Figure 6E, lanes 1, 2 and 5) or, surprisingly, eIF5B T665A (Figure 6E, lane 4) does not promote 48S IC formation on the first start codon as with wild-type eIF5B (Figure 6E, lane 3). When we substitute wild-type eIF1A for eIF1A I144A, eIF5 and wild-type eIF5B cause 48S IC assembly on the first start codon as efficiently as in the presence of wild-type eIF1A (Figure 6E, lanes 6 and 7). This result is in a good agreement with the described data (28), where yeast eIF1A I153A does not affect the translation initiation before subunit joining. As expected, eIF5 and eIF5B ΔH14 do not reveal stimulatory effect in the system with eIF1A I144A (Figure 6E, lane 8). eIF1A I144A does not inhibit the stimulation of 48S IC formation by wild-type eIF5B but compromise subunit joining by this protein, whereas, on the contrary, eIF5B T665A is able to mediate subunit joining but does not increase the 48S IC assembly. These findings indicate that functions of eIF5B in translation initiation are distinct in the 48S IC formation and subunit joining.

## DISCUSSION

Translation is one of the basic cellular processes. Although key details of this process are well understood, molecular mechanisms of many fundamental steps remain unknown. As a result, the entire set of canonical initiation factors eIFs 1, 1A, 2, 3, 4A, 4B, 4F, 40S ribosomal subunit and Met-tRNA_i_^Met^ permits the 48S IC formation in an *in vitro* reconstituted system only on the limited number of mRNAs. Recently discovered DExH box RNA helicase DHX29 expands the group of mRNAs on which 48S IC can be assembled, but the reconstitution efficiency is still much lower than in RRL. To improve the system, we employed the RRL fractionation approach and found that eIF5 and eIF5B stimulate the 48S IC formation.

Although eIF5 may act in translation initiation before and after the 48S IC assembly as a GAP for eIF2, the function of eIF5B is limited until now by subunit joining. Therefore, we show that eIF5B also reveals the activity during ribosomal scanning. Interestingly, it has been recently described that eIF5 stabilizes the binding of GDP to eIF2 and in such a way inhibits the activity of the guanine-nucleotide exchange factor eIF2B (44). Moreover, novel cryo-EM data shows that eIF5B upon ribosomal binding contacts the initiator tRNA and stabilizes it on the ribosome like a bacterial homolog IF2. Thus, it is not surprising that eIF5 and eIF5B may play multiple roles in translation process. We tested different mRNAs and found that eIF5 and eIF5B stimulate the 48S IC formation most efficiently on those mRNAs whose initiation in the reconstituted system deviates from optimal. These mRNAs include ones with structured 5′-UTR, non-optimal initiation codon context, AUG codon very close to 5′-end and near-cognate CUG start codon.

To get insights into the mechanism of stimulation, we examined the potential interplay between eIFs 5, 5B and each initiation factor presented in the system suggesting that eIF5 and eIF5B may act through the activity modulation of other proteins. The omission of eIFs 4A, 4B, 4F or DHX29 helicase from the system does not influence the stimulatory effect suggesting that eIF5 and eIF5B are not involved in the 43S PIC attachment and mRNA secondary structure unwinding. Based on the analysis of 43S PIC and 48S IC integrity after SDG centrifugation, we found that eIF5-induced hydrolysis of eIF2-bound GTP slightly reduces the affinity of eIF2 and eIF3 to the 43S PIC, but does not influence the association of eIF3 with 48S IC due to stabilizing eIF3-mRNA contacts. eIF5B does not affect the affinity of eIF2 and eIF3 to the 43S PIC. Toeprint experiments on native β-globin mRNA revealed that eIF5 and eIF5B do not change the activity of eIF1 and eIF1A. Taken together, we conclude that eIF5 and eIF5B do not impact the activity of initiation factors in the system besides the eIF5-mediated stimulation of eIF2 GTPase function.

We did not find eIF5 and eIF5B associated with the 43S PIC and 48S IC after a SDG centrifugation. eIF5 has been now confirmed as a component of MFC in yeast, plants and mammals. eIF5B forms the binary complex with eIF1A and has several binding sites on the 40S subunit. Since these proteins both mediate the stimulatory effect, they should have at least the low affinity to the ribosomal complexes. One more interesting finding is that α and β subunits of eIF2 as well as nucleotide modifications of initiator tRNA are not essential for the stimulatory effect. It is noteworthy, because α subunit of eIF2 is responsible for the recognition of a ‘−3’ key context nucleotide, whereas nucleotide modifications of initiator tRNA are necessary for the 48S IC formation on some viral RNAs.

Toeprint experiments revealed that eIF5 and eIF5B synergistically remove the aberrant +9 nt signal on a native β-globin mRNA during ribosomal scanning. Notably, while the +9 nt toeprint disappears, the full-length signal does not appear instead. This finding indicates that the aberrant 48S IC does not dissociate in this case and mRNA simply occupies the exit channel properly. Therefore, simultaneous presence of eIF5 and eIF5B in the system contributes to the proper positioning of mRNA in the mRNA binding channel of 40S subunit.

Based on the cross-linking results, eIF5 and eIF5B together cause a strong 40S/mRNA rearrangement at the ‘−3’ and ‘+4’ key context positions resulting in closer contacts of these nucleotides with rpS5 and rpS15, which are suggested to be involved in the context recognition. Therefore, such 43S PIC rearrangements may lead to the higher yield of 48S IC during ribosomal scanning. In agreement with this hypothesis, eIF5 and eIF5B do not promote the 48S IC assembly during the IRES-mediated initiation, since the mRNA placement in the 40S subunit channel and the start codon context are not essential for IRESs. However, it is equally possible that the effects seen on addition of eIF5 occur as a result of eIF2*GDP dissociation, decreasing ‘−3U’ cross-linking to eIF2α and increasing one to rpS5, which will now be the only polypeptide in proximity to ‘−3U’ in the exit channel. The increased cross-linking of ‘+4U’ to rpS15 could also reflect dissociation of eIF2*GDP and a consequent altered position of Met-tRNA_i_^Met^ in the P site.

eIF5-induced hydrolysis of eIF2-bound GTP is absolutely necessary, whereas GTPase activity of eIF5B is dispensable for the stimulatory effect. In the light of recent cryo-EM data on the ribosomal position of eIF5B, it is attractive to speculate that Met-tRNA_i_^Met^ after GTP hydrolysis or subsequent eIF2*GDP release becomes flexible. eIF5B upon ribosomal binding contacts initiator tRNA resulting in its rearrangement and stabilization in the ribosomal complex. Such a rearrangement also causes docking of the initiator tRNA and the initiation codon of mRNA favoring the 48S IC formation during scanning rather than leaky scanning. Consistently, the addition of eIF5 alone promotes a 48S IC dissociation in our toeprint experiments, whereas the simultaneous presence of eIF5B completely impedes this process. The finding that eIF5B stabilizes Met-tRNA_i_^Met^ in the absence of eIF2 during the IRES-mediated 48S IC formation on HCV-like RNAs also supports our hypothesis (45,46).

Employing the mutational analysis, we found that eIF1A I144A obstructs subunit joining but enables the stimulatory effect suggesting that the contact between eIF1 and eIF5B is critical after rather than before the 48S IC formation. Since eIF5B ΔH14 prevents both the stimulation and subunit joining, H14 is important for another process besides the contact with eIF1A. This helix is unique in eukaryotes. It is a part of domain IV, which is involved in the stabilization of Met-tRNA_i_^Met^ on the ribosome. Therefore, H14 may support Met-tRNA_i_^Met^ or provide 43S PIC rearrangement during scanning. Contrary to eIF1A I144A, eIF5B T665A supports subunit joining but impedes the stimulation. Since eIF5B adopts substantially different conformations in GTP- and GDP-bound states, mutation in the GTP-binding center should cause the conformational changes of eIF5B. Although this rearrangement does not affect the function of eIF5B in subunit joining, it is absolutely critical for the stimulation. These results are in a good agreement with recently published data that yeast eIF5B stabilizes Met-tRNA_i_^Met^ binding to 80S ICs and that this function is impaired by altering the length and rigidity of helix H12 of eIF5B connecting GTP-binding domain cup to the domain IV base (47). Thus, we conclude that functions of eIF5B in translation initiation are distinct in the 48S IC formation and subunit joining. So, there should be a switch between eIF5B functions. We believe that this switch is triggered upon the establishment of codon–anticodon interactions and mediated by the conformational changes of eIF5B or by the formation of contact between eIF1A and eIF5B after the removal of eIF1A C-terminal tail from the P site of 40S subunit.

It is well known that the carboxyl terminal domain (CTD) of eIF5 promotes 43S PIC formation stabilizing interactions among eIF1, eIF2 and eIF3 in the yeast and human MFC (3,4). However, the potential role of eIF5-CTD in ribosomal scanning and initiation codon selection was completely obscure. Based on nuclear magnetic resonance (NMR) studies, it has been recently shown that eIF5-CTD contains the evolutionary conserved, overlapping surface for the interaction with eIF2β and eIF1 (48). Mutational analysis of eIF2β binding site of eIF5-CTD revealed that the interaction between eIF2β and eIF5-CTD mediates the shift from the open to the closed scanning-arrested state of ribosomal complex. Since the interaction between eIF1 and eIF5-CTD increases the affinity of eIF1 to the 43S PIC in the open state, the establishment of contact between eIF2β and eIF5-CTD should stimulate eIF1 dissociation from 43S PIC (48,49). Another recent study discovered the novel, evolutionary conserved contact between N-terminal tail (NTT) of eIF1A and eIF5-CTD (50). NMR studies revealed that eIF1A- and eIF2β-binding sites on eIF5-CTD almost totally overlap. Mutational analysis suggested that the interaction between eIF1A-NTT and eIF5-CTD contributes to anchoring of eIF1 to 43S PIC in the open state. Therefore, eIF1A-NTT hides the eIF2β-binding site of eIF5-CTD during ribosomal scanning and in such a way keeps eIF1 in the 43S PIC (49,50). Employing mammalian reconstituted system, we found that the holo and βless forms of eIF2 contribute equally to the yield of 48S IC on both initiation codons of TC mRNA, which is consistent with the reported data in the same system (32), and to the stimulatory effect. Therefore, our results deviate from reported data that the establishment of contact between eIF2β and eIF5-CTD promotes the dissociation of eIF1 from 43S PIC (48). Unfortunately, we cannot evaluate the contribution of the contact between eIF1A-NTT and eIF5-CTD to the stimulatory effect, since all experiments were conducted in the presence of eIF1A, which is essential for the efficient reconstitution of 48S IC. Found discrepancy is not surprising. Although the contacts of eIF5-CTD with eIF2β, eIF1 and eIF1A-NTT evolutionary conserved, the initiation stage, in contrast to elongation, termination and ribosomal recycling stages, is the most divergent between yeast and mammals. In particular, the net of interactions within the MFC is different (4). Moreover, MFC establishes contacts with eIF4G via eIF5 and eIF1 in yeast and via eIF3 in mammals (49). Thus, in contrast to yeast, the interaction of eIF5-CTD with different components of mammalian MFC is probably more important for the integrity of 43S PIC rather than for ribosomal scanning and initiation codon selection.

**Figure 7.**
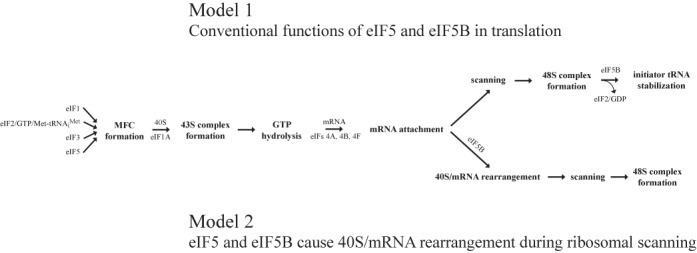
Models for eIF5 and eIF5B activities in stimulation of 48S IC formation. Model 1: eIF5 promotes 48S IC formation during ribosomal scanning by allowing GTP hydrolysis and AUG recognition to occur at the expense of continued scanning downstream. eIF5B acts only following AUG recognition and release of eIF2*GDP from the 48S IC by its ability to stabilize Met-tRNA_i_^Met^ in the P site. Model 2: eIF5 induces the hydrolysis of eIF2-bound GTP in 43S PIC. eIF5B causes the rearrangement of GDP-state 43S PIC favoring the establishment of codon–anticodon interactions upon start codon recognition.

Based on our results, we propose the following mechanism for the eIF5 and eIF5B activity in the stimulation of 48S IC formation (Figure 7). First, eIFs 1, 3, 5 and eIF2 TC form a MFC. Next, MFC and eIF1A cooperatively bind to a 40S subunit and form a 43S PIC. Then, eIF5 induces the hydrolysis of eIF2-bound GTP, but only in a part of 43S PICs. eIF5 also continues to promote the GTP hydrolysis during subsequent initiation steps. After assembly, 43S PIC attaches the 5′-end of mRNA employing eIFs 4A, 4B and 4F. For the next steps, we propose two alternative models. Model 1 is based on the conventional functions of eIF5 and eIF5B (eIF5 GAP function and role of eIF5B domain IV in stabilizing Met-tRNA_i_^Met^ in the P site following eIF2*GDP release). According to this model, after mRNA attachment, 43S PIC scans 5′-UTR of mRNA downstream to the initiation codon. GTP hydrolysis increases the probability that scanning ribosomal complexes will recognize and arrest scanning at a non-optimal start codon. Therefore, eIF5 alone may promote 48S IC formation simply by allowing GTP hydrolysis and AUG recognition to occur at the expense of continued scanning downstream. However, such 48S ICs are less stable due to eIF2*GDP dissociation from Met-tRNA_i_^Met^. eIF5B is then required to stabilize Met-tRNA_i_^Met^ in the P site. So, eIF5B may operate only following AUG recognition and release of eIF2*GDP from the 48S IC by its ability to stabilize Met-tRNA_i_^Met^ in the P site. This model is in a good agreement with recently published data that eIF5 opposes the scanning-promoting function of eIF1 at AUGs in poor context and near-cognates and thereby increases utilization of these poor initiation sites *in viv*o (51). Model 2 is more speculative. At the mRNA attachment and during the following ribosomal scanning, eIF5B binds to the ribosomal complex. Upon binding, eIF5B causes the rearrangement of a GDP-state 43S PIC favoring the establishment of codon–anticodon interactions under the initiation codon recognition. This model correlates with all our experimental data. It is particularly supported by conformational changes of ribosomal complexes as revealed by cross-linking and toeprint assays. Therefore, both alternative models are equally possible. In conclusion, although there are no *in vivo* data on the percentage of 43S PICs reaching the start codon in a GDP-state, eIF5 and eIF5B should anyway contribute to the 48S IC formation during the initiation stage.

## SUPPLEMENTARY DATA

Supplementary Data are available at NAR Online.

SUPPLEMENTARY DATA
