# eIF5 and eIF5B together stimulate 48S initiation complex formation during ribosomal scanning

**DOI:** 10.1093/nar/gkab068

**Published:** 2021-02-12

On 26 September 2014, NAR published the article ‘eIF5 and eIF5B together stimulate 48S initiation complex formation during ribosomal scanning’ by Vera P. Pisareva, and Andrey V. Pisarev (1).

At the request of the Ethical Committee at SUNY Downstate Medical Center, the Editors are publishing an Expression of Concern regarding the above article.

The Editors wish to alert the readers that questions have been raised about the validity of some of the figures presented in this article as detailed below.

*Allegations*: There are splice junctions between lanes 6/7 in Figure 2B and between lanes 7/8 in Figure 6E. The CTAG lanes have been spliced onto Figure 1B and Lane 3 appears to have been inserted between lanes 2/4 in Figure 3A.

*Ethical Committee Determination*: Authors committed research misconduct in the form of falsification. The Committee finds that failure to adhere to ethical standards evident in the extensive use of unannotated spliced images constitutes reckless behaviour on the part of the Authors. The Committee determines that the pervasive use of migration markers produced on separate gels constitutes knowing misrepresentation of data.

While these irregularities may not fundamentally affect the results and conclusions of the article, the Editors nonetheless note that such manipulations of data and corresponding figures is unacceptable.

Keith R. Fox, Barry L. Stoddard

Senior Executive Editors

## References

[1] Pisareva V.P. , Pisarev A.V. eIF5 and eIF5B together stimulate 48S initiation complex formation during ribosomal scanning. Nucleic. Acids. Res. 2014; 42:12052–12069. 25260592 10.1093/nar/gku877 PMC4231746

