# Supplementary material for: eIF5 and eIF5B together stimulate 48S initiation complex formation during ribosomal scanning
Source: Nucleic Acids Res. 2014 Sep 26;42(19):12052–69. doi: 10.1093/nar/gku877 (PMC4231746; doi:10.1093/nar/gku877)
Supplement: SUPPLEMENTARY DATA [file supp_42_19_12052__index.html]

eIF5 and eIF5B together stimulate 48S initiation complex formation during ribosomal scanning — eIF5 and eIF5B together stimulate 48S initiation complex formation during ribosomal scanning — SUPPLEMENTARY DATA 

# eIF5 and eIF5B together stimulate 48S initiation complex formation during ribosomal scanning

## SUPPLEMENTARY DATA

**Files in this Data Supplement:**

- SUPPLEMENTARY DATA
