# Supplementary material for: eIF5 and eIF5B together stimulate 48S initiation complex formation during ribosomal scanning
Source: Nucleic Acids Res. 2014 Sep 26;42(19):12052–69. doi: 10.1093/nar/gku877 (PMC4231746; doi:10.1093/nar/gku877)
Supplement: SUPPLEMENTARY DATA [file supp_gku877_nar-01531-r-2014-File009.pdf]

**SUPPLEMENTARY DATA**

**INVENTORY OF SUPPLEMENTARY DATA**

1. Supplementary materials and methods.
2. Supplementary references.

## SUPPLEMENTARY MATERIALS AND METHODS

### Plasmids

Expression vectors for $His_6$-tagged eIFs 1, 1A, 4A, 4B, 5, *E.coli* methionyl-tRNA synthetase as well as transcription vectors for Stem1 mRNA, CAA-AUG mRNA, "-3U" mRNA, "+4U" mRNA, β-globin mRNA, HCV RNA, EMCV RNA, $tRNA_i^{Met}$ have been described (19,31,32,39,52,53). Vector for the expression of human N-terminal truncated eIF5B (lacked the first 586 aa) in *E.coli* cells was prepared by cloning of protein coding region following $His_6$-tag into *NcoI/BamHI* sites of pET28a. Vectors for the expression of eIF1A I144A, eIF5B T665A and eIF5B ΔH14 were constructed by site-directed mutagenesis of eIF1A and eIF5B expression vectors. Transcription vector for CAA-CUG mRNA was made by inserting DNA flanked by a T7 promoter and an *EcoRI* restriction site into pUC57 (GenScript Corp.). Vectors for transcription of TC, AC and AG mRNAs were prepared by inserting corresponding DNA into pUC57 (GenScript Corp.). Transcription vector for eIF4H mRNA was constructed by RT-PCR from total human RNA and cloning into pUC19. mRNAs, viral RNAs and $tRNA_i^{Met}$ were transcribed using T7 RNA polymerase. All mRNA transcripts were capped with Vaccinia virus capping enzyme (NEB) according to manufacturer's protocol. 5'-end 32P-labelling of β-globin mRNA was performed with Vaccinia virus capping enzyme and [α-32P]GTP. For toeprint assay, 4-thioU (TriLink Biotech) was introduced in "-3U" mRNA co-transcriptionally. Radiolabelled "-3U" and "+4U" mRNAs containing 4-thioU were obtained by transcription in the presence of [α-32P]CTP and 4-thioU.

### Aminoacylation of tRNA

*In vitro* transcribed $tRNA_i^{Met}$ and rabbit native total tRNAs (Promega) were aminoacylated with methionine in the presence of recombinant *E.coli* methionyl-tRNA synthetase as described (19). For methionyl-puromycin assay, *in vitro* transcribed $tRNA^{Met}_i$ was aminoacylated using 35S-labelled methionine (5 x $10^5$ cpm/pmol).

### eIF5 and eIF5B purification

Native eIF5 and eIF5B were purified from the 40-50% ammonium sulfate (AS) precipitation fraction of the 0.5 M KCl ribosomal salt wash (RSW) that was prepared from 450 ml of RRL. The fraction was dialyzed against buffer B (20 mM Tris-HCl, pH 7.5, 0.1 mM EDTA, 2 mM

DTT, 10% glycerol) + 100 mM KCl and applied to a diethylaminoethyl cellulose (DEAE) column equilibrated with buffer B + 100 mM KCl. Both proteins were eluted at 100-250 mM KCl. This fraction was dialyzed against buffer B + 100 mM KCl and applied to a phosphocellulose (P-11) column equilibrated with buffer B + 100 mM KCl. eIF5 and eIF5B were eluted at 400-800 mM KCl, dialyzed against buffer B + 100 mM KCl and applied to a FPLC MonoQ 5/50 GL column. Fractions were collected across a 100-500 mM KCl gradient. eIF5 eluted at ~360 mM KCl and eIF5B- at 480mM KCl.

Recombinant eIF5B was expressed in 1 L of *E.coli* BL21(DE3) after induction by 0.1 mM IPTG for 16 h at 16$^{o}$C and isolated by affinity chromatography on Ni-NTA agarose followed by FPLC on MonoQ column. Fractions were collected across a 30-500 mM KCl gradient. eIF5B elutes at 100mM KCl.

**Purification of eIF1A and eIF5B mutants**

Recombinant eIF1A I144A, eIF5B T665A and eIF5B ΔH14 were expressed in 1 L of *E.coli* BL21(DE3) after induction by 0.1 mM IPTG for 16 h at 16$^{o}$C and isolated by affinity chromatography on Ni-NTA agarose followed by FPLC on MonoQ column. Fractions were collected across a 100-500 mM KCl gradient for eIF1A mutant and a 30-500 mM KCl gradient for eIF5B mutants. eIF1A I144A, eIF5B T665A and eIF5B ΔH14 elute at 310 mM, 70 mM and 100 mM KCl, respectively.

**Assembly of initiation complexes**

To reconstitute 43S PIC and 48S IC, we incubated different combinations of 0.2 pmol DHX29, 5 pmol eIFs 2/3/4F, 10 pmol eIFs 1/1A/4A/4B, 5 pmol native or 10 pmol recombinant eIF5 and eIF5B, 6 pmol Met-tRNA$_i^{Met}$, and 1.75 pmol 40S ribosomal subunit in a 20 μl reaction mixtures containing buffer A (20 mM Tris-HCl, pH 7.5, 100 mM KCl, 2.5 mM $MgCl_2$, 0.1 mM EDTA, 1 mM DTT) with 0.2 mM ATP and 0.2 mM GTP/GMPPNP for 5 min at 37$^{o}$C, supplemented the reaction mixture with 0.3 pmol mRNA or viral RNA, and additionally incubated for 10 min at 37$^{o}$C. To form 80S IC, 48S IC was supplemented with 10 pmol each recombinant eIF5 and eIF5B (if they were not added before), 2.5 pmol 60S ribosomal subunit, and incubated for 10 min at 37$^{o}$C.

**Toeprint assay**

We estimated the yield of 48S IC assembled on different mRNAs and viral RNAs employing toeprint assay as described (19). This assay involves the extension by the reverse transcriptase of the radiolabelled primer annealed to the mRNA. Generally, ribosomal complexes yield toeprint signals at the leading edge of the 40S subunits at the position +16-+18 nt downstream of mRNA triplet in the P site. Signals are visualized after the electrophoresis of cDNA in the polyacrylamide gel and autoradiography.

**GTPase assay**

eIF2 TC, 43S PIC and 48S IC assembled in the presence of 0.33μM [γ-32P]GTP as well as [γ-32P]GTP in a free form in the same concentration were incubated in a 20 μl reaction mixtures

containing buffer A with/without 10 pmol eIFs 1/1A/5/5B, 5pmol eIF3, 1.75 pmol 40S subunits, 2.5 pmol 80S ribosomes, and 10 pmol $(CUUU)_9$ RNA for 15 min at 37°C, and subjected to PEI cellulose chromatography as described (19).

**Purification and analysis of ribosomal complexes by sucrose density gradient centrifugation**

For GTPase assay, 43S PIC assembled in the presence of 0.33μM [γ-32P]GTP in a 400 μl reaction mixture with scaled amounts of initiation components was purified by centrifugation through 10-30% SDG prepared in buffer A in a Beckman SW55 rotor at 53,000 rpm for 75 min. To examine the effect of eIF5 and eIF5B on the composition of 43S PIC and 48S IC, ribosomal complexes reconstituted in a 400 μl reaction mixture with scaled amounts of initiation components were subjected to centrifugation through 10-30% SDG prepared in buffer A in a Beckman SW55 rotor at 53,000 rpm for 75 min. Fractions that corresponded to ribosomal complexes were resolved in SDS-PAGE and stained with Sypro Ruby Red (Invitrogen) or assayed by immunobloting with antibodies against eIF2α (Abcam).

**Methionyl-puromycin assay**

To compare the activity of 48S IC in the formation of 80S IC, methionyl-puromycin assay was employed. 48S ICs were reconstituted on CAA-CUG mRNA with 35S-labelled Met-$tRNA_i^{Met}$ in the presence/absence of eIFs 5/5B, supplemented with 60S subunits and eIFs 5/5B (if they were not added before), and additionally incubated for 10 min at 37°C. After assembly, ribosomal complexes were treated with 1 mM puromycin for 40 min at 37°C and extracted with ethyl acetate. 35S-labelled methionyl-puromycin formation was measured by scintillation counting of ethyl acetate extract.

**UV cross-linking assay**

To examine the effect of eIF5 and eIF5B on 43S PIC rearrangement, we performed UV cross-linking experiments. 48S ICs were reconstituted with/without eIFs 5/5B on co-transcriptionally 32P-labelled "-3U" and "+4U" mRNAs containing 4-thioU at -3 and +4 key context positions, respectively. To keep the integrity of ribosomal complexes, we omitted the purification step by SDG centrifugation. After assembly, 48S ICs were irradiated at 360 nm for 30 min on ice using a UV-Stratalinker (Stratagene) and digested with 5 units RNAse A for 10 min at 37°C. To identify cross-linked proteins, ribosomal complexes were assayed by SDS-PAGE and autoradiography.

**Analysis of eIF1A and eIF5B mutants activity in 80S IC formation**

To test the activity of mutants, 80S ICs were assembled on 32P-labelled β-globin mRNA with eIF1A I144A, eIF5B T665A, eIF5B ΔH14 or their wild-type forms in a 400 μl reaction mixture with scaled amounts of initiation components. Next, ribosomal complexes were resolved by centrifugation through 10-30% SDG prepared in buffer A in a Beckman SW55 rotor at 53,000 rpm for 105 min. To assign ribosomal peaks, optical density of gradient fractions was measured at 260 nm. The yield of 48S IC and 80S IC was determined by Cherenkov counting of

incorporated radiolabelled mRNA in gradient fractions containing 40S subunits and 80S ribosomes, respectively.

## SUPPLEMENTARY REFERENCES


52. Berry, K.E., Waghray, S. and Doudna, J.A. (2010) The HCV IRES pseudoknot positions the initiation codon on the 40S ribosomal subunit. *RNA*, **16**, 1559-1569.
53. Pisarev, A.V., Skabkin, M.A., Pisareva, V.P., Skabkina, O.V., Rakotondrafara, A.M., Hentze, M.W., Hellen, C.U. and Pestova, T.V. (2010) The role of ABCE1 in eukaryotic posttermination ribosomal recycling. *Mol. Cell*, **37**, 196-210.
